# Comparative performance of multiple-list estimators of key population size

**DOI:** 10.1371/journal.pgph.0000155

**Published:** 2022-03-10

**Authors:** Steve Gutreuter

**Affiliations:** Division of Global HIV and TB, U.S. Centers for Disease Control and Prevention, Atlanta, Georgia, United States of America

## Abstract

Estimates of the sizes of key populations (KPs) affected by HIV, including men who have sex with men, female sex workers and people who inject drugs, are required for targeting epidemic control efforts where they are most needed. Unfortunately, different estimators often produce discrepant results, and an objective basis for choice is lacking. This simulation study provides the first comparison of information-theoretic selection of loglinear models (LLM-AIC), Bayesian model averaging of loglinear models (LLM-BMA) and Bayesian nonparametric latent-class modeling (BLCM) for estimation of population size from multiple lists. Four hundred random samples from populations of size 1,000, 10,000 and 20,000, each including five encounter opportunities, were independently simulated using each of 30 data-generating models obtained from combinations of six patterns of variation in encounter probabilities and five expected per-list encounter probabilities, producing a total of 36,000 samples. Population size was estimated for each combination of sample and sequentially cumulative sets of 2–5 lists using LLM-AIC, LLM-BMA and BLCM. LLM-BMA and BLCM were quite robust and performed comparably in terms of root mean-squared error and bias, and outperformed LLM-AIC. All estimation methods produced uncertainty intervals which failed to achieve the nominal coverage, but LLM-BMA, as implemented in the dga R package produced the best balance of accuracy and interval coverage. The results also indicate that two-list estimation is unnecessarily vulnerable, and it is better to estimate the sizes of KPs based on at least three lists.

## Introduction

Among the 1.7 million new HIV infections globally in 2018, 54% occurred among key populations (KPs), particularly female sex workers (FSW), people who inject drugs (PWID), men who have sex with men (MSM), transgender women, clients of sex workers, and sex partners of other KP members [1]. Even in the generalized HIV epidemics in eastern and southern Africa where 75% of new infections occurred among the general population, targeted scale-up of antiretroviral therapy and other interventions among KPs may be the most efficient way to avert new infections [2, 3]. For those reasons, provision of HIV services to KPs has long been an important component of the (United States) President’s Emergency Plan for AIDS Relief [4] and The Global Fund to Fight AIDS, Tuberculosis and Malaria [5]. Scaling and targeting of life-saving HIV services to KPs, and evaluating the efficacy of those services requires knowledge about the sizes of KPs [6].

KP members are often adversely affected by discrimination and stigma [7]. Stigma and criminalization [8] create incentives for key population members to remain hidden, which challenges both population size estimation (PSE) and provision of HIV services. Therefore multiple methods of PSE have been recommended [9]. PSE based on the method known by the monikers “capture-recapture” and the “multiplier method” is a statistically principled approach which has been widely used to estimate the sizes of KPs [10–20]. Such multiple-list PSE is commonly based on only two lists, but three-or-more-list estimation [21–24] is becoming increasingly common.

Ratio estimation of population sizes from partial observations from two lists (sources or sampling events) dates to 1786 [25], and later became known as “capture-recapture” or “mark-recapture” estimation among animal ecologists [26, 27]. Although early applications and developments focused heavily on non-human animal populations, the methods have been applied more broadly including human birth registration [28], census undercount [29], and epidemiological applications [30–32] which trace back to at least 1968 [33]. “Multiplier” or “service-multiplier” estimation in the public-health literature [34–37] is a rediscovery of ratio estimation of population sizes. The essential data are counts of population members that are recorded on two lists (sources), wherein individuals on the first list can be defined as “marked” and those on the second list are tabulated as either previously encountered (“recaptured”) or newly encountered. Estimation from two lists requires the strong assumptions that 1) the population is static over the observation interval, 2) previously encountered individuals are identified without error, 3) individuals are sampled independently, and 4) all population members share a common and constant probability of encounter. The first assumption is well-approximated by sampling over short time intervals. The second and third assumptions remain uncertain in KPs because humans can choose whether or not interview or to disclose a previous encounter. The fourth assumption is untenable for KPs; it is inconceivable that all KP members share a common and constant probability of encounter. Rather, we should expect that individual KP members are highly inhomogeneous in their encounter probabilities.

Subsequent statistical developments included accommodation of more than two encounter sources (survey rounds or service rosters) [38], which enables relaxation of the fourth assumption via the development of model-based estimation using distribution mixtures [39, 40] and loglinear models (LLMs) of observation frequencies [41] or encounter probabilities [42]. Multiple variations of LLMs which satisfy different assumptions about inhomogeneities in encounter probabilities are commonly fitted to the data, and then the “best” model by some criterion (usually Akaike’s Information Criterion, AIC) is selected for estimation of *N*. That conventional approach is henceforth denoted LLM-AIC. Unfortunately, two or more LLM variations can fit data equally well and yet produce very different estimates and uncertainty intervals [43].

Discrepant estimates occur because the population-size parameter *N* is generally not identified [40, 44, 45]. Roughly, a parameter is said to be unidentified whenever its true value remains unknown given an infinite number of observations. Recognition of the unidentifiability of PSE parameters seems absent from the epidemiological and public-health literature, yet it has enormous implications for estimation. The lower bound of population size [46] is identified [47], but is rarely the desired target for KPs.

More recent Bayesian developments eliminate the need for model selection and may improve robustness. Bayesian model averaging of loglinear models (LLM-BMA) reduces the volatility resulting from choice of a single model by properly accounting for model uncertainty [48]. The feasible set of LLMs are fitted and *N* is estimated as the model-probability-weighted average from those LLMs. Bayesian nonparametric latent-class modeling (BLCM) [49] abandons the LLM framework in favor of estimation from distribution mixtures. Consider that—with sufficient information—a population that is inhomogeneous with respect to encounter probability could be correctly stratified into some potentially large number of homogeneous classes. It that case, a well-informed distribution mixture could be employed to estimate *N*. However the number of homogeneous classes is unknown in practice. Instead, BLCM “learns” the most probable latent classes from the data in a Bayesian way, and prevents over-parameterization by imposing a parsimonious prior distribution.

Public-health scientists who estimate the sizes of KPs need to know the performances of alternative estimation methods in order to make informed choices. To obtain a more objective basis for choice, LLM-AIC, LLM-BMA and BLCM were compared using simulated populations of known size and different patterns of variation in encounter probability. Secondarily, the frequencies with which LLM-AIC correctly matches the underlying data-generating models were quantified, and the performance of selected heterogeneity corrections in LLM-AIC estimation were compared.

## Materials and methods

### Study design

The numbers of population members in simulated samples from known populations were estimated using LLM-AIC, LLM-BMA and BLCM. The population sizes, inhomogeneities in encounter probabilities and the number of observation events/lists were varied in the simulated samples to assess the effects of those factors on PSE. The simulated data enabled comparison among methods based on their abilities to estimate the true population size.

### Sample simulation

Four hundred random samples from populations of size *N* = 1,000, 10,000 and 20,000, each including five encounter opportunities, were independently simulated from each combination of six models of variation in encounter probabilities *p*, and five expected per-list encounter probabilities E(*p*), producing a total of 36,000 samples from which to estimate *N* based on 2–5 lists. The population sizes were chosen to align with KP size estimates, which commonly fall in the range of 10^3^–10^4^, and 20,000 was a compromise for computational feasibility in simulations.

The patterns in encounter probability are standards from the literature [50–52]. The choice of inhomogeneity patterns was a simplification for comparative purposes; the patterns in KPs may be nearly infinite. The “heterogeneity” model $M_{h}$ accommodates encounter probabilities which vary among individuals. Humans are capable of complex behaviors and preferences, including variations in propensities to seek social and sexual contacts, attend particular venues, or seek services from organizations through which encounters may be listed. Therefore we should not expect KP members to share a common encounter probability. The “temporal” model $M_{t}$ allows encounter probabilities to vary over lists/times. Encounter probabilities might vary with many temporal factors including, weather, economic conditions, day-of-the week, and variations in law-enforcement efforts. The assumption that KP members have temporally constant encounter probability is extreme and risks biased estimation. The “behavioral” model $M_{b}$ imposes a common expected probability of first encounter on each individual and—after the first encounter—that individual’s encounter probability is henceforth increased or decreased. For this study, the expected encounter probabilities were reduced by 50% after the first. Behavioral effects can arise when, for example, the first contact tends to be either pleasing or displeasing to KP members. For example, FSW might seek out subsequent contacts with surveyors if the “mark” (typically a uniquely identifiable gift) received during their first contact was perceived to be desirable. Conversely, MSM and PWID might avoid subsequent contacts with recognizable surveyors in order to minimize their risk of recrimination or prosecution. Given the complexity of human behavior, we should anticipate combinations of all three basic patterns of inhomogeneity. Models $M_{th}$, $M_{bh}$ and $M_{tbh}$ are combinations of $M_{h}$, $M_{t}$ and $M_{b}$.

**Fig 1.**
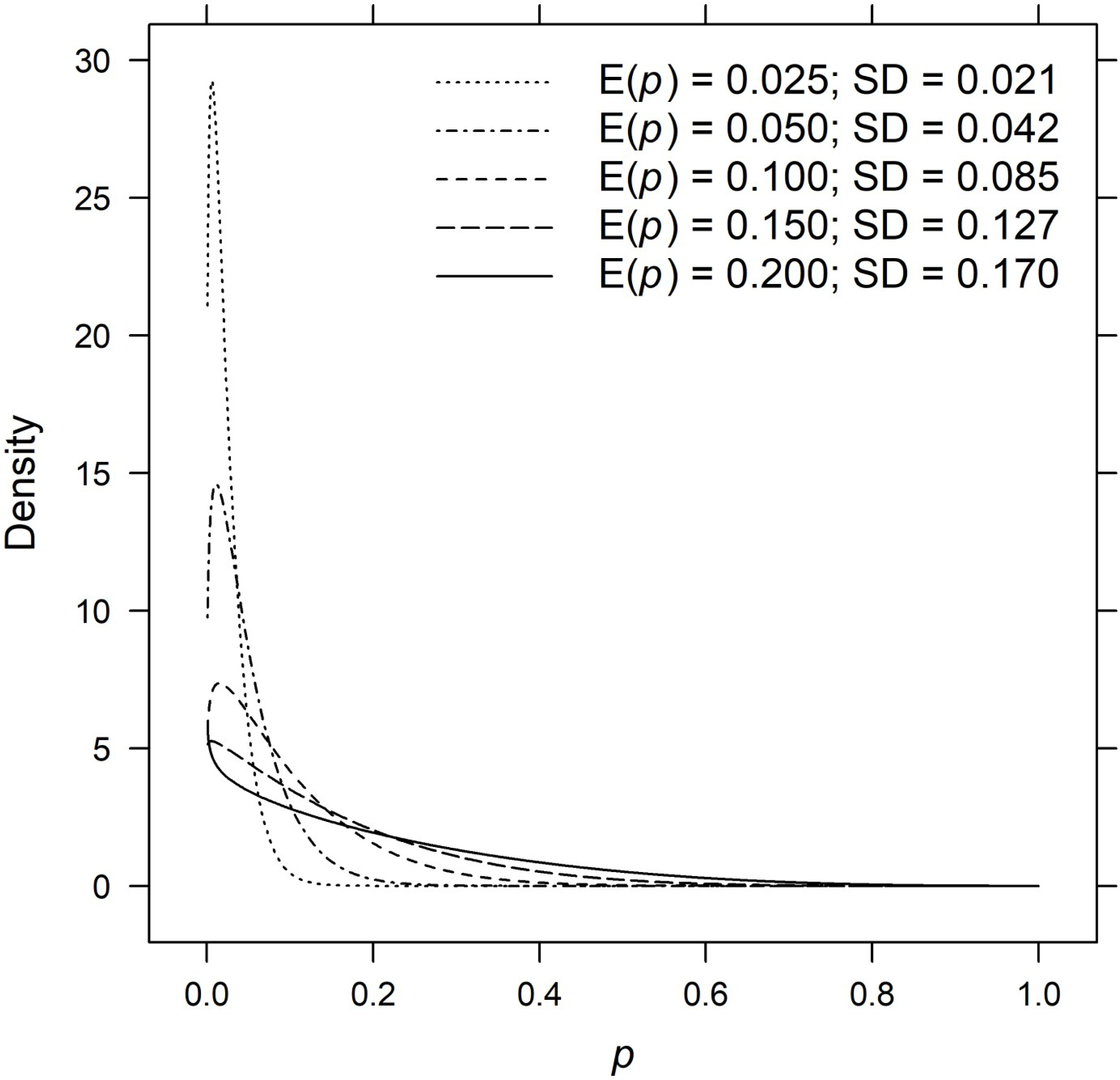
Beta densities for encounter probabilities. Beta densities for list-wise encounter probabilities from data-generating models $M_{h}$, $M_{b}$, $M_{t}M_{th}$, $M_{bh}$ and $M_{bht}$.

Individual encounter histories were simulated from beta-Bernoulli distributions given by *y*_*ijk*_ ~ Bernoulli(*p*_*ijk*_) and *p*_*ijk*_ ~ Beta(*θ*_*ijk*_), where *p*_*ijk*_ denotes the encounter probability for sample *i*, *i* = 1, . . ., 400, individual *j*, *j* = 1, . . ., *N* and list *k*, *k* = 1, . . ., 5. The *θ*_*ijk*_ are 2 × 1 vectors of shape parameters (*β*_1_, *β*_2_) (Table 1), which were chosen to produce expected encounter probabilities E(*p*) = 0.025, 0.050, 0.100, 0.150 and 0.200 given a coefficient of variation of 0.85. The inhomogeneities in encounter probabilities, as measured by the standard deviation of the Beta distribution, ranged by more than a factor of eight from 0.021 to 0.170 (Fig 1). The complete encounter history for individual *j* in sample *i* and lists 1, . . ., *k* is the *k*-element vector *y*_*ijk*_ of zeros and ones, wherein a one in position *k* indicates that the individual appears on list *k* and a zero indicates absence. Given a total of *K* lists, there are 2^*K*^ − 1 observable encounter histories and one unobservable history consisting entirely of zeros. The unobservable encounter histories were removed from the simulated data prior to estimation.

Given encounter probability *p* and *k* lists of encounters, the proportion of population members observed for the first time from list *k* is given by *p*_1_(*k*) = *p*(1 − *p*)^*k*−1^, which has expectation with respect to the Beta distribution

$$\text{E}\left[p_{1}(k)\right]=\frac{\Gamma \left(\beta_{1}+\beta_{2}\right)\Gamma \left(\beta_{1}+1\right)\Gamma \left(\beta_{2}+k+1\right)}{\Gamma \left(\beta_{1}\right)\Gamma \left(\beta_{2}\right)\Gamma \left(\beta_{1}+\beta_{2}+k\right)},$$

where Γ(･) denotes the Gamma function, and *β*_1_ and *β*_2_ are the shape parameters of the Beta distribution (S1 Text). Therefore the expected percentages of the populations observed at least once ranged from 4.9% for two lists with E(*p*) = 0.025, to 54.7% from five lists with E(*p*) = 0.200 (Table 1). That may encompass the most likely range of sampling percentages from encounters within KPs affected by HIV. For example, sampling encountered approximately 10%, 22% and 30% of the estimated sizes of the MSM, PWID and FSW populations, respectively, in Kampala, Uganda [22].

### Estimation

The population-size parameter *N* was estimated from each combination of estimation method, sample replicate, data-generating model and sequentially cumulative sets of *K* = 2, . . ., 5 lists. The first estimation method was traditional LLM-AIC estimation as implemented in the Rcapture package [53] for R [54]. This traditional application of model selection to multiple-list population-size estimation ignores model uncertainty. The Rcapture package is comprehensive, and was used only to implement estimation of models $M_{b}$, $M_{t}$, $M_{h}$, $M_{bh}$ and $M_{th}$. Models $M_{bt}$ and $M_{bth}$ are not loglinear; the latter cannot be fitted using Rcapture, and estimation of the former is unstable and was ignored in this study. Fitted models were compared using AIC, and the model having the smallest AIC was selected for estimation of *N*.

The Rcapture package enables use of alternative heterogeneity corrections in models $M_{h}$, $M_{bh}$ and $M_{th}$. Use of more than one heterogeneity correction is problematic because estimates can vary substantially among the correction methods and yet share a common AIC, leaving the analyst without any objective basis for choice. Estimates from the “Poisson2” heterogeneity correction for models $M_{h}$, $M_{bh}$ and $M_{th}$ were used for comparison in this simulation study, per the demonstration of superiority in S1 Table.

The second method was LLM-BMA [48], as implemented in the dga R package [55], which accounts for model uncertainty. The dga package is currently limited to 3–5-list sampling. The set of feasible estimation models is a large superset of our data-generating models, and increases geometrically in size with the number of lists included in the estimation. Each feasible model and model probabilities are computed for each. The final PSE estimate is the probability-weighted average of model-specific estimates. The prior maximum number of unobserved population members was set to 10*N*, based on the premise that the true size of KPs might be known within an order of magnitude. The hyperparameter for the hyper-Dirichlet prior on list intersection probabilities was set to 2^−*K*^, where *K* = 3, . . ., 5 denotes the number of lists included in the estimation, as recommended by the package authors. A brief sensitivity analysis of the prior specification is presented in S1 Text. Estimation of *N* is based on Laplace approximation, which nonetheless becomes computationally time-consuming with increasing *K* because of the large number of feasible models.

Last, population size was estimated using Bayesian nonparametric latent-class modeling [49], (BLCM) as implemented in the LCMCR R package. The value for the maximum number of latent classes was set to 10. The prior distribution for the vector of latent-class probabilities is a stick-breaking formulation of a Dirichlet process prior having parameter *α*. That prior concentrates the probability mass on the first few latent classes to avoid overfitting. The hyperprior for *α* is a Gamma distribution having parameters *a* and *b*, which were both set to 0.25 to provide a reasonably vague specification for the simulations [49]. A brief sensitivity analysis of the prior specification is presented in S1 Text. Estimation is based on Markov Chain Monte Carlo (MCMC) simulation. Based on a preliminary analysis, pre-convergence “burn-in” samples of 500,000 iterations were discarded and the posterior sample consisted of an additional 50,000 iterations out of 5,000,000 after thinning by 100 to reduce autocorrelation. In practice, far fewer burn-in iterations are typically required. The numbers chosen here assured convergence and stable estimation of posterior quantiles with small Monte Carlo error.

The resulting LLM-AIC, LLM-BMA and BLCM estimates ${\hat{N}}_{i}$ were compared using estimated root mean-squared error ($\hat{\text{RMSE}}=\sqrt{\frac{1}{m}\sum_{i=1}^{m}{\left(N-{\hat{N}}_{i}\right)}^{2}}$, $\mathrm{bias}\left(=\text{E}\left[{\hat{N}}_{i}\right]-N\right)$ and the estimated coverage probabilities of uncertainty intervals (95% profile-likelihood confidence intervals for LLM-AIC, and Pr = 0.95 credible intervals for LLM-BMA and BLCM). Mean-squared error is the sum of sampling variance and squared bias, and is an omnibus measure of accuracy and precision of estimation. LLM-AIC, LLM-BMA and BLCM estimates were compared over the aggregated set of data-generating models in order to assess estimation of real populations, for which the underlying data-generating processes are never known.

Finally, the unreliability of LLM-AIC to correctly match underlying data-generating models $M_{h}$, $M_{t}$, $M_{b}$, $M_{bh}$ and $M_{th}$ was evaluated to illustrate a consequence of unidentified parameters. All computations were performed using R 4.0.3 [54]. R code and population-size estimates are provided in S1 File.

## Results

### Comparative performance of LLM-AIC, LLM-BMA and BLCM estimation

**Fig 2.**
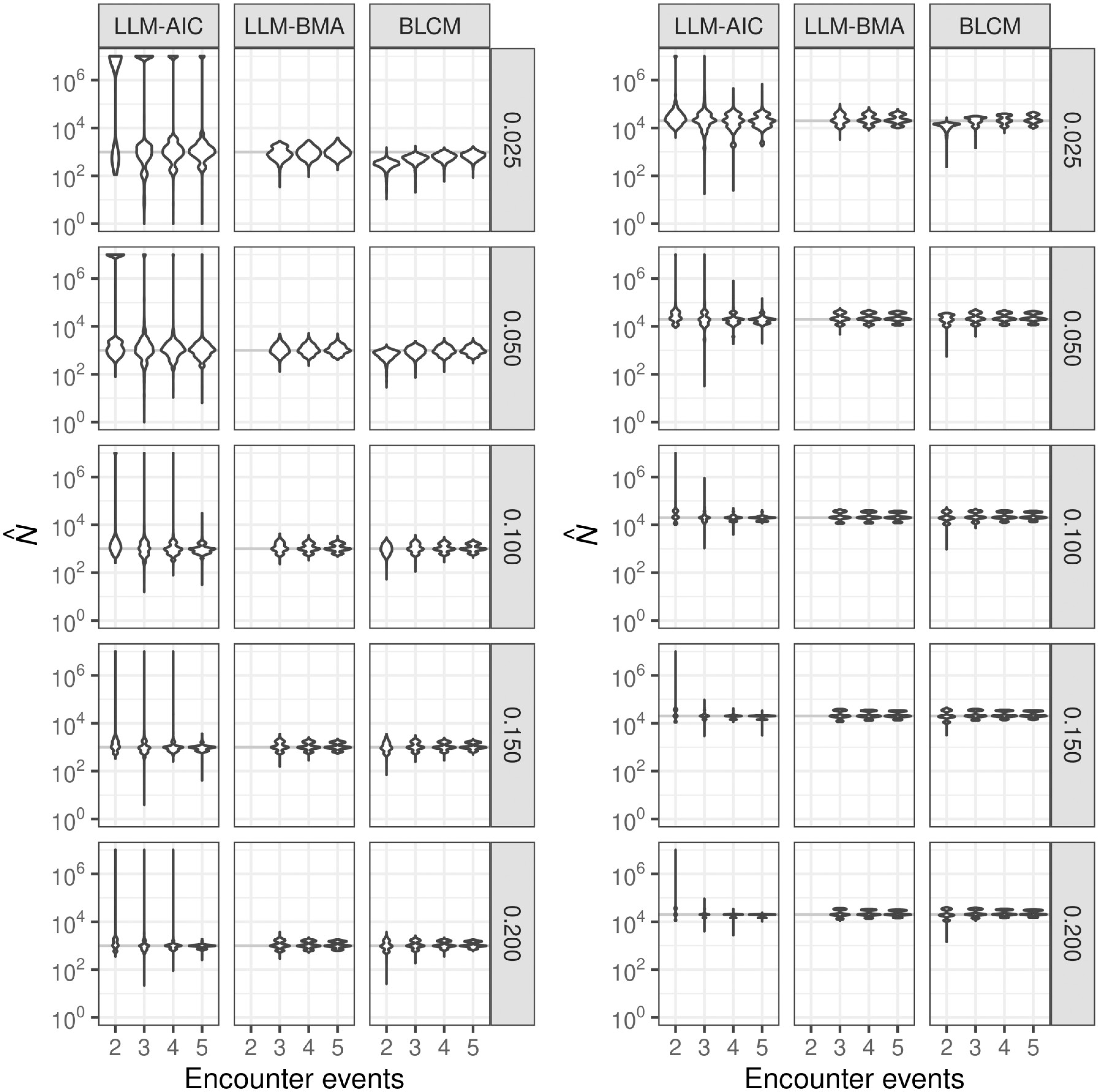
Distributions of estimates the sizes of populations consisting of 1,000 (left) and 20,000 (right) members. Estimation was based on 2–5 encounter event-histories generated from models $M_{h}$, $M_{b}$, $M_{t}$, $M_{bh}$, $M_{ht}$, and $M_{bht}$ for expected encounter probabilities of 0.025, 0.050, 0.100, 0.150 and 0.200 (right margins). LLM-AIC denotes selection of the single best LLM based on AIC, LLM-BMA denotes Bayesian model-averaging of loglinear models, and BLCM denotes nonparametric Bayesian latent-class model estimation. Estimates larger than 10^7^ are heaped at that value. Results for *N* = 10, 000 were similar to those for *N* = 20, 000.

Population-size estimates from all methods exhibited at least some evidence of multiple modes across expected encounter probabilities and numbers of encounter events over the mix of data-generating models (Fig 2). The LLM-AIC estimates exhibited the largest ranges, usually spanning more than seven orders of magnitude. The distributions of LLM-AIC estimates were reasonably compact for estimating populations of 1,000 only where the per event expected encounter probability was 0.2 over five sampling events. LLM-BMA and BLCM modeling performed nearly equally, but BLCM estimation produced distributions having longer lower tails. LLM-BMA and BLCM estimation outperformed LLM-AIC estimation in terms of both root mean-squared error (RMSE) and bias (Table 2). The estimated RMSEs and bias of the LLM-AIC estimates were effectively infinite for all combinations of population size and expected encounter probability when estimating from two lists, and estimates sometimes exceeded 10^19^, which is a manifestation of unidentified parameters. LLM-AIC estimation became moderately reliable in terms of RMSE and bias from three-event sampling only where the expected per-event encounter probabilities were at least 0.150. In contrast, RMSEs and bias from both BLCM and LLM-BMA estimation indicated that those methods produced estimates within the correct order of magnitude across all expected encounter probabilities and three or more sampling events, and BLCM estimation produced similarly reasonable estimates from two sampling events.

Uncertainty intervals from LLM-AIC and BLCM estimation almost always failed to achieve nominal coverage, and produced intervals which were too narrow (Table 3). In contrast, the credible intervals from LLM-BMA estimation tended to be too wide, with coverage probabilities frequently larger than 0.98.

**Fig 3.**
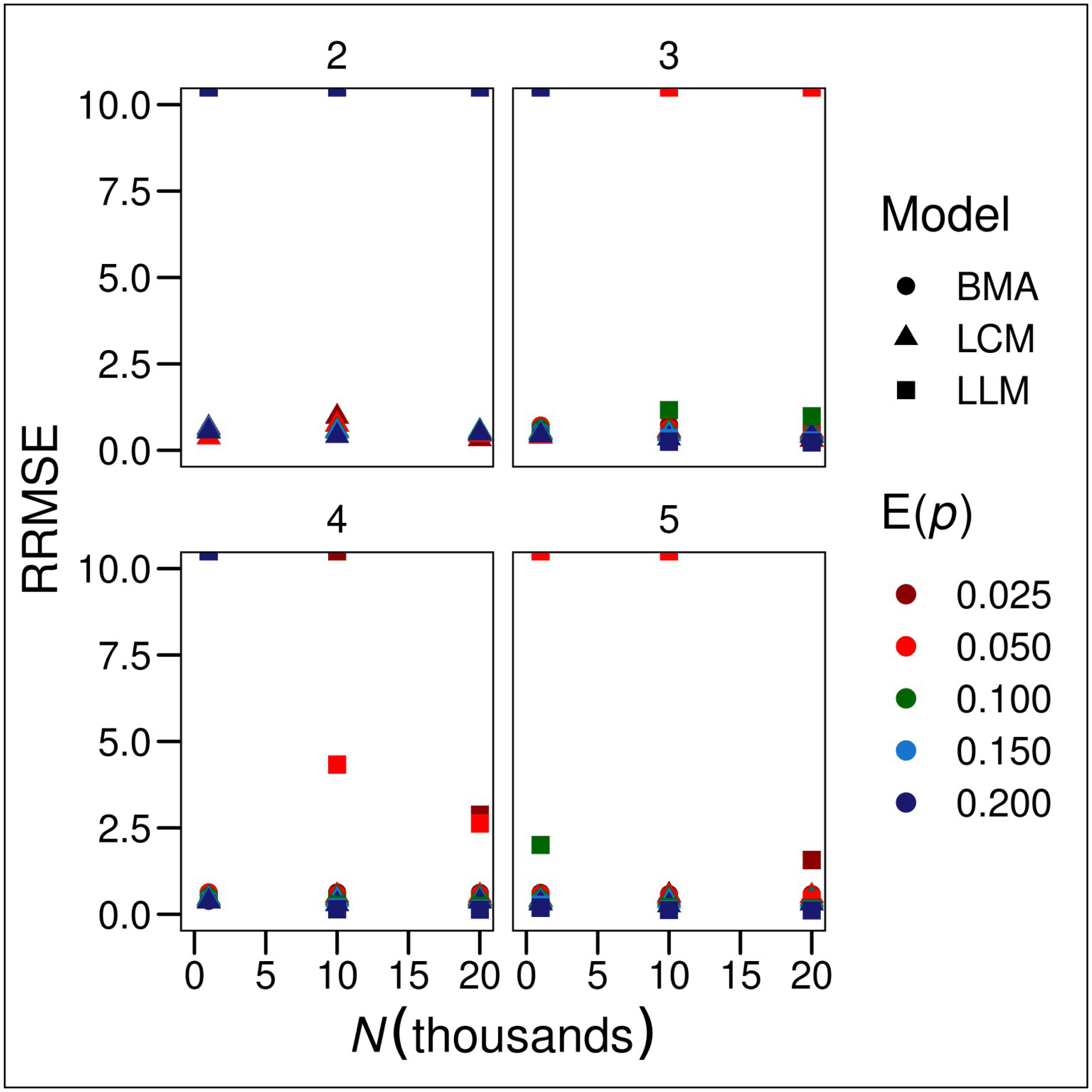
Relative root mean-squared errors (RRMSE = RMSE ÷ *N*) of estimators of population size. LLM-AIC denotes loglinear model selection, LLM-BMA denotes Bayesian model-averaging of loglinear models, BLCM denotes nonparametric Bayesian latent-class model estimation and E(*p*) denotes expected encounter probability.

Relative RMSE was largely constant across true population sizes (Fig 3), indicating that the results of of this study apply at least over the range of simulated population sizes. Relative RMSE was also independent of the expected encounter probabilities.

### Ability of LLM-AIC to match the data-generating models

Loglinear model selection offers the hope of inferring the type of variation in encounter probabilities. For example, if the AIC-best fitting model happens to be $M_{h}$, then one might hope that encounter probabilities differed among individuals, but not over time, and similarly there would be no behavioral effect. That would be more than one should hope for because the parameter *N* is almost always unidentified. The simulation results provide a concrete illustration of the consequences of unidentifiability. No more than 8.1% of the replicate data sets generated by $M_{th}$ and no more than 24.8% of the replicates generated by $M_{bh}$ were correctly identified by the AIC-best model in populations of size 1,000 and 20,000 (Table 4). Correct matching of $M_{b}$ and $M_{h}$ data and models increased with increasing expected detection probability and, less distinctly, with the number of sampling lists. Matchings by data-generating model are shown in S2 Table.

## Discussion

PSE is inherently challenging, and especially for KPs affected by discrimination, prosecution and stigma. Unlike inanimate or non-human population members, people can refuse contact and acceptance/disclosure of marks. For key populations those marks are typically inexpensive small gifts or membership on some service list. It is unreasonable to expect that all people will share a common and constant propensity seek services from a particular entity, or to accept interpersonal contact and marks, and to disclose prior receipt of a mark. Therefore $M_{h}$ may be the simplest plausible form of inhomogeneity among KPs, and more complex forms than those used in these simulations may be in play. For example, some KP members might increase their encounter probability after the first contact if they find the gift marks attractive while others might decrease their subsequent encounter probabilities, leading to distribution mixtures of different behavioral effects.

A priori, the analyst confronting PSE has no knowledge of patterns of variation in encounter probabilities. Worse, the lack of identifiability of model parameters [44, 45] precludes the possibility of reliable inference about the form of inhomogeneity, as is clearly illustrated by the results of this study. Therefore the analyst can never be confident that any model matches the underlying data-generating process. Model uncertainty is especially problematic where the estimates differ substantially, which is often the case. The only practical recourse is to use estimation methods which are robust to model uncertainty and inhomogeneities in encounter probabilities.

LLM-BMA and BLCM estimation demonstrated considerable robustness. Both generally outperformed LLM-AIC in terms of sample RMSE and bias, except where per-list encounter probabilities were at least 0.1. Two-list LLM-AIC estimation—which has been commonplace for PSE—was unreliable across all three population sizes and all expected encounter probabilities. LLM-BMA and BLCM estimates were generally comparable and never produced effectively infinite RMSEs. RMSEs decreased with increasing numbers of lists across all three methods, which should be unsurprising given that the observed fraction of a population increases with the number of lists.

All three PSE methods failed to achieve the nominal 95% coverage for uncertainty intervals in these simulations. LLM-AIC and BLCM estimation produced intervals with substantially less than the nominal coverage, while LLM-BMA estimation, as implemented in the dga package for R, produced highly conservative intervals. Overall, LLM-BMA estimation tended to produce the best balance of accuracy and interval coverage in this study.

The dga package for R is convenient for loglinear model averaging, but other options are available with greater effort. For example, frequentist model averaging has been proposed [56], but was not considered here because it requires custom coding by the analyst. Likely more important, frequentist model averaging lacks the theoretical grounding of BMA and does not exploit prior information on *N*, so that practically infinite estimates are not precluded. In practice, some upper bound of convenience on *N* is always known. For example, the number of FSW and cannot be larger than the female population, and the number of MSM is highly unlikely to be more than 10% of the male population in most settings [57, 58]. Therefore the ability to constrain the upper bound on *N* in the prior for Bayesian model averaging as implemented in the dga package is an advantage.

The limitations of this study arise from reliance on Monte Carlo simulation, which provides weaker conclusions than formal mathematical proof. However, simulation is the only practical way to compare estimates with known population sizes. Monte Carlo simulation relies on machine-generated pseudo-random numbers, and therefore results will vary slightly across different streams of random numbers. All results from this simulation study are conditional on the choice of data-generating models, and also on control and prior parameters for the estimation models. The choice of data-generating models was broad and representative of commonly expected patterns of variation in encounter probability, but was not exhaustive. Results may differ from other data-generating models, other control and prior parameters for estimation, and other true population sizes and numbers of lists. Still, this simulation study provides the first cross-cutting comparison of the performance distinctly different PSE methods, and provides an objective basis for choice among those methods.

## Conclusion

The results of this simulation study strongly suggest that some form of comprehensive model averaging or latent-class modeling should be the default choice for PSE, and that estimation should be based on data from at least three encounter events or lists. The two Bayesian approaches, LLM-BMA and BLCM, were more robust than LLM-AIC. LLM-BMA, as implemented in the freely available dga R package is particularly appealing because the analyst will almost always have some prior information on population size. Although none of the methods produced uncertainty intervals that achieved nominal coverage, the conservative intervals produced by LLM-BMA, as implemented in the dga R package, came closest in these simulations.

All of the estimation methods compared in this study are implemented using the freely available R packages. However, they are also easily accessible to those unfamiliar with R via web-based Multiple Source Recapture web application at https://www.epiapps.com/.

## Supplementary Material

S1 Table **S1 Table. Comparison of LLM-AIC heterogeneity corrections.** Sample sizes and estimated root mean-squared errors $\left(\hat{\text{RMS}E}\right)$ from Poisson2, Darroch and Gamma3.5 heterogeneity corrections in loglinear estimation models $M_{h}$ and $M_{th}$ for estimation of population size N from five lists generated from models $M_{h}$, $M_{bh}$, $M_{th}$, and $M_{bth}$.

S1 Text **S1 Text. Supplemental methods and results.** A PDF file containing the derivation of the probability of first encounters in succesive lists and brief sensitivity analyses of the prior distributions for LLM-BMA and BLCM estimation.

S2 Table **S2 Table. Frequencies (percentages) by which individual data-generating models were matched to various AIC-best estimation models.** The column labels are self-explanatory. This table is an expansion of Table 3 showing matchings by each data-generating model. In practice, the analyst would not know the data-generating model.

S1 File **S1 File. R code and population-size estimates.** A zip archive containing the R code used to generate the random samples from the simulated populations and to estimate population size from those samples. The population size estimates are also included because obtaining those from the samples is computationally burdensome.

## Figures and Tables

**Table 1.** Shape parameters *β*_1_ and *β*_2_ for the data-generating Beta distributions, expected encounter probabilities E(p), and expected proportions population members encountered for the first time on list *k* = 1, . . ., 5, E[*p*_1_ (*k*)].

*β*_1_	*β*_2_	E(p)	*k*	E[*p*_1_ (*k*)]	Cumulative E[*p*_1_ (*k*)]
1.3245	51.6548	0.025	1	0.025	0.025
			2	0.024	0.049
			3	0.023	0.072
			4	0.022	0.094
			5	0.021	0.115
1.2649	24.0327	0.050	1	0.050	0.050
			2	0.046	0.096
			3	0.042	0.138
			4	0.039	0.176
			5	0.036	0.212
1.1457	10.3111	0.100	1	0.100	0.100
			2	0.083	0.183
			3	0.070	0.252
			4	0.059	0.312
			5	0.051	0.363
1.0265	5.8167	0.150	1	0.150	0.150
			2	0.111	0.261
			3	0.086	0.347
			4	0.068	0.415
			5	0.055	0.470
0.9073	3.6291	0.200	1	0.200	0.200
			2	0.131	0.331
			3	0.093	0.424
			4	0.069	0.493
			5	0.054	0.547

**Table 2.** Root mean-squared error (RMSE) and bias of estimators of the sizes *N* of simulated populations.

*N*	Performance measure	Expected Pr (encounter)^1^	Number of encounters or lists										
			2		3			4			5		
			LLM-AIC	BLCM	LLM-AIC	LLM-BMA	BLCM	LLM-AIC	LLM-BMA	BLCM	LLM-AIC	LLM-BMA	BLCM
1,000	RMSE	0.025	> 10^9^	692	> 10^9^	543	506	> 10^9^	557	423	> 10^9^	618	376
		0.050	> 10^9^	404	> 10^9^	716	390	> 10^9^	633	424	> 10^9^	572	417
		0.100	> 10^9^	491	> 10^9^	630	541	> 10^9^	531	478	2,005	466	432
		0.150	> 10^9^	566	> 10^9^	537	502	> 10^9^	452	433	276	396	381
		0.200	> 10^9^	508	> 10^9^	464	447	> 10^9^	389	373	190	335	322
	Bias	0.025	> 10^9^	−680	> 10^9^	−31	−468	< −10^9^	44	−356	> 10^9^	109	−279
		0.050	> 10^9^	−307	> 10^9^	220	−42	> 10^9^	194	26	> 10^9^	175	56
		0.100	> 10^9^	55	> 10^9^	251	179	> 10^9^	203	163	−22	173	148
		0.150	> 10^9^	135	> 10^9^	203	182	> 10^9^	162	158	−38	136	138
		0.200	> 10^9^	120	< −10^9^	165	164	> 10^9^	128	139	−49	102	115
10,000	RMSE	0.025	> 10^9^	4,215	> 10^9^	7,386	2,874	> 10^9^	6,303	4,316	> 10^9^	5,794	5,334
		0.050	> 10^9^	3,533	> 10^9^	6,173	5,626	43,319	5,551	5,355	> 10^9^	5,294	5,155
		0.100	> 10^9^	5,336	11,655	5,318	5,246	2,872	4,860	4,777	2,092	4,520	4,423
		0.150	> 10^9^	4,953	3,603	4,766	4,701	2,002	4,235	4,179	1,544	3,798	3,751
		0.200	> 10^9^	3,929	2,511	4,290	3,432	1,479	3,662	2,948	1,178	3,141	2,583
	Bias	0.025	> 10^9^	−3,932	> 10^9^	2,888	28	> 10^9^	2,496	1,644	> 10^9^	2,333	2,457
		0.050	> 10^9^	117	> 10^9^	2,663	2,275	−391	2,386	2,229	> 10^9^	2,264	2,171
		0.100	> 10^9^	1,733	71	2,265	2,290	−269	2,001	2,091	−321	1,820	1,945
		0.150	> 10^9^	1,557	−19	1,901	2,084	−173	1,685	1,880	−326	1,475	1,691
		0.200	> 10^9^	182	−6	1,639	829	−294	1,407	763	−475	1,162	643
20,000	RMSE	0.025	> 10^9^	8,679	> 10^9^	13,859	6,127	57,543	12,382	9,052	31,361	11,618	10,649
		0.050	> 10^9^	7,622	> 10^9^	11,758	11,443	52,505	11,007	10,828	8,080	10,548	10,418
		0.100	> 10^9^	10,366	19,729	10,395	10,300	4,825	9,640	9,480	3,736	8,971	8,791
		0.150	> 10^9^	9,731	5,732	9,413	9,326	3,285	8,345	8,294	2,650	7,490	7,489
		0.200	> 10^9^	9,188	4,558	8,473	8,479	2,646	7,191	7,274	2,270	6,112	6,254
	Bias	0.025	> 10^9^	−7,897	> 10^9^	5,791	524	−613	5,277	3,121	1,275	4,964	4,267
		0.050	> 10^9^	1,214	> 10^9^	5,284	5,035	−836	4,883	4,781	−194	4,621	4,598
		0.100	> 10^9^	3,447	574	4,434	4,643	−188	4,012	4,308	−388	3,670	4,065
		0.150	> 10^9^	3,117	50	3,772	4,186	−370	3,436	3,886	−729	3,037	3,557
		0.200	> 10^9^	2,671	23	3,318	3,769	−707	2,830	3,359	−972	2,340	2,859

1 For first encounters in data-generating models $M_{b}$, $M_{bh}$ and $M_{tbh}$.

LLM-AIC denotes selection of the AIC-best loglinear model, LLM-BMA denotes Bayesian model-averaging of loglinear models, and BLCM denotes nonparametric Bayesian latent-class model estimation.

**Table 3.** Coverage of uncertainty intervals (95% confiddence intervals for loglinear model selection LLM-AIC, and Pr = 0.95) credible intervals for Bayesian model averaging LLM-BMA and latent-class modeling BLCM).

*N*	Expected Pr(encounter)^1^	Number of encounters or lists										
		2		3			4			5		
		LLM-AIC	BLCM	LLM-AIC	LLM-BMA	BLCM	LLM-AIC	LLM-BMA	BLCM	LLM-AIC	LLM-BMA	BLCM
1,000	0.025	0.975	0.201	0.693	1.000	0.658	0.699	1.000	0.687	0.687	1.000	0.688
	0.050	0.831	0.832	0.627	1.000	0.828	0.559	1.000	0.756	0.508	1.000	0.682
	0.100	0.688	0.853	0.444	1.000	0.588	0.501	1.000	0.493	0.509	0.999	0.475
	0.150	0.496	0.836	0.498	0.997	0.503	0.562	0.992	0.479	0.604	0.982	0.459
	0.200	0.422	0.821	0.541	0.982	0.497	0.620	0.963	0.475	0.663	0.937	0.449
10,000	0.025	0.771	0.514	0.498	1.000	0.761	0.456	1.000	0.478	0.402	1.000	0.412
	0.050	0.492	0.803	0.400	1.000	0.463	0.464	1.000	0.465	0.490	1.000	0.455
	0.100	0.403	0.818	0.525	1.000	0.472	0.599	1.000	0.469	0.615	1.000	0.469
	0.150	0.383	0.827	0.588	0.996	0.484	0.629	0.990	0.478	0.703	0.983	0.474
	0.200	0.362	0.792	0.626	0.982	0.582	0.729	0.964	0.579	0.761	0.940	0.572
20,000	0.025	0.651	0.537	0.423	1.000	0.505	0.405	1.000	0.445	0.422	1.000	0.446
	0.050	0.407	0.795	0.460	1.000	0.446	0.480	1.000	0.442	0.554	1.000	0.437
	0.100	0.376	0.807	0.573	1.000	0.457	0.580	1.000	0.454	0.596	1.000	0.449
	0.150	0.374	0.823	0.590	0.998	0.475	0.629	0.993	0.461	0.682	0.984	0.441
	0.200	0.346	0.826	0.641	0.985	0.487	0.730	0.965	0.465	0.737	0.950	0.450

1 For first encounters in data-generating models $M_{b}$, $M_{bh}$ and $M_{tbh}$.

**Table 4.** Percentages of correct matchings of the data-generating model by the AIC-best LLM population size estimation models based on overlap of 3–5 lists for each of four per-list expected probabilities of encounter. See text for explanation of the data-generating models.

*N*	Expected Pr(Encounter)^1^	Lists	Data-generating Model				
			$M_{b}$	$M_{bh}$	$M_{h}$	$M_{th}$	$M_{t}$
1,000	0.025	3	55.7	19.6	14.5	0.0	52.8
		4	42.1	22.2	9.9	0.0	78.7
		5	51.8	24.8	13.4	0.0	90.2
	0.050	3	51.1	12.3	30.0	5.5	59.9
		4	36.9	21.5	40.0	1.7	82.1
		5	55.9	19.8	39.4	3.3	86.9
	0.100	3	64.1	10.6	53.9	3.7	69.2
		4	45.3	10.5	65.9	2.7	83.0
		5	56.2	9.7	79.4	4.2	87.4
	0.150	3	59.9	8.2	66.8	3.8	71.8
		4	69.1	10.8	82.9	3.3	78.8
		5	74.2	13.4	90.5	1.5	84.7
	0.200	3	64.0	7.1	75.6	8.1	73.7
		4	73.9	10.8	87.5	4.5	84.9
		5	77.6	10.2	91.8	6.0	83.9
20,000	0.025	3	51.1	15.8	53.5	3.7	76.7
		4	52.9	14.8	47.9	5.2	83.8
		5	50.5	12.9	65.9	5.3	83.5
	0.050	3	67.0	7.3	63.6	6.3	81.2
		4	64.6	12.0	78.0	5.0	82.5
		5	71.9	12.3	89.4	2.0	84.0
	0.100	3	75.5	4.0	88.5	5.0	79.7
		4	73.4	4.8	89.0	2.0	85.2
		5	75.8	1.5	91.0	2.6	85.0
	0.150	3	70.2	3.5	88.5	6.5	81.5
		4	76.5	1.2	93.2	1.9	84.2
		5	80.0	0.0	95.2	3.3	84.2
	0.200	3	73.8	2.5	87.0	3.0	82.5
		4	82.0	0.0	88.0	5.1	82.8
		5	83.5	0.0	91.8	4.8	83.0

1 For first encounters in $M_{b}$, $M_{bh}$ and $M_{tbh}$.

## Data Availability

All data needed to replicate all of the figures, graphs, tables, statistics, and other values are provided within Supporting Information S1 File.
