# Supplementary material for: Comparative performance of multiple-list estimators of key population size
Source: PLOS Glob Public Health. 2022 Mar 10;2(3):e0000155. doi: 10.1371/journal.pgph.0000155 (PMC9345571; doi:10.1371/journal.pgph.0000155)
Supplement: S1 Table — Sample sizes and estimated root mean-squared errors (RMSE^) from Poisson2, Darroch and Gamma3.5 heterogeneity corrections in loglinear estimation models Mh and Mth for estimation of population size N from five lists generated from models Mh, Mbh, Mth, and Mbth. (PDF) [file pgph.0000155.s002.pdf]

Table S2. Sample sizes and estimated root mean-squared errors ($\widehat{\text{RMSE}}$) from Poisson2, Darroch and Gamma3.5 heterogeneity corrections[1] in loglinear estimation models $\mathcal{M}_h$ and $\mathcal{M}_{th}$ for estimation of population size N from five lists generated from models $\mathcal{M}_h$, $\mathcal{M}_{bh}$, $\mathcal{M}_{th}$, and $\mathcal{M}_{bth}$. A total of $n = 1,600$ estimates were available for each combination of estimation model, encounter probability and number of encounter events, but those for which variance estimation failed were excluded. The Poisson2 heterogeneity correction produced the smallest $\widehat{\text{RMSE}}$s in all but two of 24 comparisons in the simulated data in fits to models $\mathcal{M}_{bh}$ and $\mathcal{M}_{tbh}$. The Darroch and Gamma3.5 corrections occasionally produced $\widehat{\text{RMSE}}$s that were more than one order of magnitude larger than the Poisson2 correction. Conversely, the Gamma3.5 correction produced the largest $\widehat{\text{RMSE}}$s in all but two comparisons.

	Estimation	Expected	Poisson2		Darroch		Gamma3.5	
N	model	Pr(encounter)[2]	n	$\widehat{\text{RMSE}}$	n	$\widehat{\text{RMSE}}$	n	$\widehat{\text{RMSE}}$
1,000	$\mathcal{M}_h$	0.025	366	1,667,192	365	$> 10^9$	339	$> 10^9$
		0.050	1,055	671	1,055	2,257	1,051	7,536
		0.100	1,562	434	1,562	635	1,560	1,061
		0.150	1,600	364	1,600	449	1,599	633
		0.200	1,600	292	1,600	294	1,600	368
	$\mathcal{M}_{th}$	0.025	366	1,614,888	365	$> 10^9$	341	$> 10^9$
		0.050	1,055	668	1,055	2,256	1,051	7,568
		0.100	1,562	433	1,562	635	1,560	1,064
		0.150	1,600	363	1,600	449	1,599	636
		0.200	1,600	291	1,600	294	1,600	371
20,000	$\mathcal{M}_h$	0.025	1,363	13,259	1,363	27,030	1,363	53,402
		0.050	1,599	9,664	1,599	12,554	1,599	17,513
		0.100	1,600	7,851	1,600	8,198	1,600	9,691
		0.150	1,600	6,680	1,600	6,478	1,600	7,291
		0.200	1,600	5,720	1,600	5,178	1,600	5,663
	$\mathcal{M}_{th}$	0.025	1,363	13,252	1,363	27,030	1,363	53,421
		0.050	1,599	9,658	1,599	12,554	1,599	17,522
		0.100	1,600	7,839	1,600	8,197	1,600	9,711
		0.150	1,600	6,662	1,600	6,477	1,600	7,325
		0.200	1,600	5,699	1,600	5,175	1,600	5,707

[1] See: Baillargeon S, Rivest L. Rcapture: Loglinear models for capture-recapture in R. Journal of Statistical Software. 2007;19(5):1–31. doi:10.18637/jss.v019.i05.

[2] For first encounters in data-generating models $\mathcal{M}_{bh}$ and $\mathcal{M}_{tbh}$.
