# Supplementary material for: Comparative performance of multiple-list estimators of key population size
Source: PLOS Glob Public Health. 2022 Mar 10;2(3):e0000155. doi: 10.1371/journal.pgph.0000155 (PMC9345571; doi:10.1371/journal.pgph.0000155)
Supplement: S2 Table — The column labels are self-explanatory. This table is an expansion of Table 3 showing matchings by each data-generating model. In practice, the analyst would not know the data-generating model. (PDF) [file pgph.0000155.s003.pdf]

Table S4. Frequencies (percentages) of data-generating models which were correctly matched by the AIC-best log-linear models.

N	Expected Pr(Encounter)	Data-generating model	Lists	AIC-best estimating model $\mathcal{M}_b$	$\mathcal{M}_{bh}$	$\mathcal{M}_h$	$\mathcal{M}_{ht}$	$\mathcal{M}_t$
1,000	0.025	$\mathcal{M}_b$	3	49	17	1	0	21
				(16.1)	(5.6)	(0.3)	(0.0)	(6.9)
			4	45	31	5	0	26
				(14.8)	(10.2)	(1.6)	(0.0)	(8.5)
			5	57	27	0	0	26
				(18.7)	(8.9)	(0.0)	(0.0)	(8.5)
		$\mathcal{M}_{bh}$	3	51	18	3	1	19
				(16.5)	(5.8)	(1.0)	(0.3)	(6.1)
			4	57	26	8	0	26
				(18.4)	(8.4)	(2.6)	(0.0)	(8.4)
			5	53	25	3	0	20
				(17.1)	(8.1)	(1.0)	(0.0)	(6.5)
		$\mathcal{M}_h$	3	56	19	16	5	14
				(16.3)	(5.5)	(4.7)	(1.5)	(4.1)
			4	46	38	12	0	25
				(13.4)	(11.1)	(3.5)	(0.0)	(7.3)
			5	37	35	15	4	21
				(10.8)	(10.2)	(4.4)	(1.2)	(6.1)
		$\mathcal{M}_{ht}$	3	62	26	3	0	15
				(19.0)	(8.0)	(0.9)	(0.0)	(4.6)
			4	48	40	2	0	25
				(14.7)	(12.3)	(0.6)	(0.0)	(7.7)
			5	46	32	1	0	26
				(14.1)	(9.8)	(0.3)	(0.0)	(8.0)
		$\mathcal{M}_t$	3	56	103	0	4	182
				(5.1)	(9.4)	(0.0)	(0.4)	(16.7)
			4	16	49	0	13	289
				(1.5)	(4.5)	(0.0)	(1.2)	(26.5)
			5	7	26	0	4	342
				(0.6)	(2.4)	(0.0)	(0.4)	(31.3)
	0.050	$\mathcal{M}_b$	3	45	16	11	0	16
				(14.2)	(5.0)	(3.5)	(0.0)	(5.0)
			4	41	31	9	3	27
				(12.9)	(9.8)	(2.8)	(0.9)	(8.5)
			5	66	28	1	1	22
				(20.8)	(8.8)	(0.3)	(0.3)	(6.9)
		$\mathcal{M}_{bh}$	3	62	16	24	4	24
				(14.2)	(3.7)	(5.5)	(0.9)	(5.5)
			4	70	31	17	4	22
				(16.1)	(7.1)	(3.9)	(0.9)	(5.0)

Continued on next page

Table S4 continued...

N	Expected Pr(Encounter)	Data-generating model	Lists	AIC-best estimating model $\mathcal{M}_b$	$\mathcal{M}_{bh}$	$\mathcal{M}_h$	$\mathcal{M}_{ht}$	$\mathcal{M}_t$
			5	76	32	27	6	21
				(17.4)	(7.3)	(6.2)	(1.4)	(4.8)
		$\mathcal{M}_h$	3	43	19	39	3	26
				(8.9)	(3.9)	(8.0)	(0.6)	(5.4)
			4	48	27	70	10	20
				(9.9)	(5.6)	(14.4)	(2.1)	(4.1)
			5	48	28	71	7	26
				(9.9)	(5.8)	(14.6)	(1.4)	(5.4)
		$\mathcal{M}_{ht}$	3	45	21	20	6	17
				(11.8)	(5.5)	(5.3)	(1.6)	(4.5)
			4	45	34	21	2	19
				(11.8)	(8.9)	(5.5)	(0.5)	(5.0)
			5	36	32	66	5	11
				(9.5)	(8.4)	(17.4)	(1.3)	(2.9)
		$\mathcal{M}_t$	3	36	95	0	20	226
				(3.1)	(8.2)	(0.0)	(1.7)	(19.4)
			4	8	35	0	27	321
				(0.7)	(3.0)	(0.0)	(2.3)	(27.6)
			5	2	12	0	38	345
				(0.2)	(1.0)	(0.0)	(3.3)	(29.6)
	0.100	$\mathcal{M}_b$	3	93	19	13	1	19
				(14.5)	(3.0)	(2.0)	(0.2)	(3.0)
			4	105	34	53	5	35
				(16.4)	(5.3)	(8.3)	(0.8)	(5.5)
			5	149	47	26	13	30
				(23.2)	(7.3)	(4.0)	(2.0)	(4.7)
		$\mathcal{M}_{bh}$	3	128	25	44	14	24
				(15.0)	(2.9)	(5.2)	(1.6)	(2.8)
			4	165	29	34	19	28
				(19.4)	(3.4)	(4.0)	(2.2)	(3.3)
			5	222	33	37	17	32
				(26.1)	(3.9)	(4.3)	(2.0)	(3.8)
		$\mathcal{M}_h$	3	34	15	97	10	24
				(4.6)	(2.0)	(13.1)	(1.3)	(3.2)
			4	36	21	162	12	15
				(4.9)	(2.8)	(21.8)	(1.6)	(2.0)
			5	21	15	251	21	8
				(2.8)	(2.0)	(33.8)	(2.8)	(1.1)
		$\mathcal{M}_{ht}$	3	48	21	75	6	14
				(10.5)	(4.6)	(16.4)	(1.3)	(3.1)
			4	40	29	59	4	17
				(8.8)	(6.4)	(12.9)	(0.9)	(3.7)

Continued on next page

Table S4 continued...

N	Expected Pr(Encounter)	Data-generating model	Lists	$\mathcal{M}_b$	$\mathcal{M}_{bh}$	$\mathcal{M}_h$	$\mathcal{M}_{ht}$	$\mathcal{M}_t$
				AIC-best estimating model				
			5	43	27	49	6	18
				(9.4)	(5.9)	(10.7)	(1.3)	(3.9)
		$\mathcal{M}_t$	3	27	65	0	28	269
				(2.3)	(5.5)	(0.0)	(2.4)	(22.8)
			4	2	13	0	52	328
				(0.2)	(1.1)	(0.0)	(4.4)	(27.8)
			5	0	4	0	46	347
				(0.0)	(0.3)	(0.0)	(3.9)	(29.4)
	0.150	$\mathcal{M}_b$	3	142	22	37	9	27
				(15.1)	(2.3)	(3.9)	(1.0)	(2.9)
			4	228	36	29	10	27
				(24.3)	(3.8)	(3.1)	(1.1)	(2.9)
			5	276	47	9	7	33
				(29.4)	(5.0)	(1.0)	(0.7)	(3.5)
		$\mathcal{M}_{bh}$	3	213	28	27	31	41
				(19.0)	(2.5)	(2.4)	(2.8)	(3.6)
			4	229	42	22	57	38
				(20.4)	(3.7)	(2.0)	(5.1)	(3.4)
			5	224	53	12	82	25
				(19.9)	(4.7)	(1.1)	(7.3)	(2.2)
		$\mathcal{M}_h$	3	27	16	157	24	11
				(2.7)	(1.6)	(15.9)	(2.4)	(1.1)
			4	13	6	290	37	4
				(1.3)	(0.6)	(29.4)	(3.8)	(0.4)
			5	2	1	362	35	0
				(0.2)	(0.1)	(36.8)	(3.6)	(0.0)
		$\mathcal{M}_{ht}$	3	43	13	55	5	15
				(11.1)	(3.4)	(14.2)	(1.3)	(3.9)
			4	36	17	50	4	14
				(9.3)	(4.4)	(12.9)	(1.0)	(3.6)
			5	38	15	65	2	15
				(9.8)	(3.9)	(16.8)	(0.5)	(3.9)
		$\mathcal{M}_t$	3	14	50	0	47	283
				(1.2)	(4.2)	(0.0)	(4.0)	(23.8)
			4	1	11	0	72	313
				(0.1)	(0.9)	(0.0)	(6.1)	(26.3)
			5	0	1	0	60	337
				(0.0)	(0.1)	(0.0)	(5.0)	(28.3)
	0.200	$\mathcal{M}_b$	3	206	32	27	24	33
				(18.6)	(2.9)	(2.4)	(2.2)	(3.0)
			4	286	42	5	13	41
				(25.8)	(3.8)	(0.5)	(1.2)	(3.7)

Continued on next page

Table S4 continued...

N	Expected Pr(Encounter)	Data-generating model	Lists	AIC-best estimating model $\mathcal{M}_b$	$\mathcal{M}_{bh}$	$\mathcal{M}_h$	$\mathcal{M}_{ht}$	$\mathcal{M}_t$
			5	309	50	0	9	30
				(27.9)	(4.5)	(0.0)	(0.8)	(2.7)
		$\mathcal{M}_{bh}$	3	233	28	4	68	59
				(19.5)	(2.3)	(0.3)	(5.7)	(4.9)
			4	201	43	1	128	27
				(16.9)	(3.6)	(0.1)	(10.7)	(2.3)
			5	138	41	0	210	11
				(11.6)	(3.4)	(0.0)	(17.6)	(0.9)
		$\mathcal{M}_h$	3	17	8	264	59	1
				(1.5)	(0.7)	(23.0)	(5.1)	(0.1)
			4	1	1	349	48	0
				(0.1)	(0.1)	(30.4)	(4.2)	(0.0)
			5	0	0	367	33	0
				(0.0)	(0.0)	(32.0)	(2.9)	(0.0)
		$\mathcal{M}_{ht}$	3	47	23	54	13	24
				(10.1)	(4.9)	(11.6)	(2.8)	(5.2)
			4	50	26	48	7	23
				(10.8)	(5.6)	(10.3)	(1.5)	(4.9)
			5	42	24	46	9	29
				(9.0)	(5.2)	(9.9)	(1.9)	(6.2)
		$\mathcal{M}_t$	3	12	46	1	45	291
				(1.0)	(3.9)	(0.1)	(3.8)	(24.5)
			4	0	4	0	56	337
				(0.0)	(0.3)	(0.0)	(4.7)	(28.3)
			5	0	0	0	64	333
				(0.0)	(0.0)	(0.0)	(5.4)	(28.0)
20,000	0.025	$\mathcal{M}_b$	3	67	13	29	3	19
				(14.5)	(2.8)	(6.3)	(0.7)	(4.1)
			4	74	31	12	2	21
				(16.1)	(6.7)	(2.6)	(0.4)	(4.6)
			5	96	40	29	1	24
				(20.8)	(8.7)	(6.3)	(0.2)	(5.2)
		$\mathcal{M}_{bh}$	3	74	24	30	7	17
				(11.6)	(3.8)	(4.7)	(1.1)	(2.7)
			4	94	32	57	10	23
				(14.7)	(5.0)	(8.9)	(1.6)	(3.6)
			5	142	35	56	18	21
				(22.2)	(5.5)	(8.8)	(2.8)	(3.3)
		$\mathcal{M}_h$	3	37	13	77	3	14
				(7.2)	(2.5)	(14.9)	(0.6)	(2.7)
			4	50	17	79	5	14
				(9.7)	(3.3)	(15.3)	(1.0)	(2.7)

Continued on next page

Table S4 continued...

N	Expected Pr(Encounter)	Data-generating model	Lists	AIC-best estimating model $\mathcal{M}_b$	$\mathcal{M}_{bh}$	$\mathcal{M}_h$	$\mathcal{M}_{ht}$	$\mathcal{M}_t$
			5	37	12	137	9	13
				(7.2)	(2.3)	(26.5)	(1.7)	(2.5)
		$\mathcal{M}_{ht}$	3	39	21	19	4	25
				(8.7)	(4.7)	(4.2)	(0.9)	(5.6)
			4	45	20	108	10	8
				(10.0)	(4.5)	(24.1)	(2.2)	(1.8)
			5	47	22	60	8	13
				(10.5)	(4.9)	(13.4)	(1.8)	(2.9)
		$\mathcal{M}_t$	3	14	66	0	13	306
				(1.2)	(5.5)	(0.0)	(1.1)	(25.5)
			4	0	14	0	51	335
				(0.0)	(1.2)	(0.0)	(4.3)	(27.9)
			5	0	0	0	66	334
				(0.0)	(0.0)	(0.0)	(5.5)	(27.9)
	0.050	$\mathcal{M}_b$	3	134	23	14	5	24
				(15.8)	(2.7)	(1.7)	(0.6)	(2.8)
			4	186	41	31	11	19
				(21.9)	(4.8)	(3.7)	(1.3)	(2.2)
			5	259	58	10	9	24
				(30.5)	(6.8)	(1.2)	(1.1)	(2.8)
		$\mathcal{M}_{bh}$	3	204	23	43	26	19
				(18.6)	(2.1)	(3.9)	(2.4)	(1.7)
			4	240	46	21	50	27
				(21.9)	(4.2)	(1.9)	(4.6)	(2.5)
			5	236	49	5	91	17
				(21.5)	(4.5)	(0.5)	(8.3)	(1.5)
		$\mathcal{M}_h$	3	27	13	133	15	21
				(2.9)	(1.4)	(14.4)	(1.6)	(2.3)
			4	23	8	256	34	7
				(2.5)	(0.9)	(27.7)	(3.7)	(0.8)
			5	3	1	346	35	2
				(0.3)	(0.1)	(37.4)	(3.8)	(0.2)
		$\mathcal{M}_{ht}$	3	50	19	47	9	17
				(11.6)	(4.4)	(10.9)	(2.1)	(3.9)
			4	51	19	45	7	17
				(11.8)	(4.4)	(10.4)	(1.6)	(3.9)
			5	55	31	42	3	20
				(12.7)	(7.2)	(9.7)	(0.7)	(4.6)
		$\mathcal{M}_t$	3	2	40	0	33	324
				(0.2)	(3.3)	(0.0)	(2.8)	(27.0)
			4	0	2	0	68	330
				(0.0)	(0.2)	(0.0)	(5.7)	(27.5)

Continued on next page

Table S4 continued...

N	Expected Pr(Encounter)	Data-generating model	Lists	$\mathcal{M}_b$	$\mathcal{M}_{bh}$	$\mathcal{M}_h$	$\mathcal{M}_{ht}$	$\mathcal{M}_t$
				AIC-best estimating model				
			5	0	0	0	64	336
				(0.0)	(0.0)	(0.0)	(5.3)	(28.0)
	0.100	$\mathcal{M}_b$	3	292	32	1	31	31
				(24.6)	(2.7)	(0.1)	(2.6)	(2.6)
			4	293	56	0	18	32
				(24.7)	(4.7)	(0.0)	(1.5)	(2.7)
			5	303	64	0	9	24
				(25.5)	(5.4)	(0.0)	(0.8)	(2.0)
		$\mathcal{M}_{bh}$	3	183	16	0	144	57
				(15.2)	(1.3)	(0.0)	(12.0)	(4.8)
			4	106	19	0	265	10
				(8.8)	(1.6)	(0.0)	(22.1)	(0.8)
			5	40	6	0	354	0
				(3.3)	(0.5)	(0.0)	(29.5)	(0.0)
		$\mathcal{M}_h$	3	3	3	345	38	1
				(0.3)	(0.3)	(29.0)	(3.2)	(0.1)
			4	0	0	356	44	0
				(0.0)	(0.0)	(29.9)	(3.7)	(0.0)
			5	0	0	364	36	0
				(0.0)	(0.0)	(30.6)	(3.0)	(0.0)
		$\mathcal{M}_{ht}$	3	39	17	65	7	13
				(8.9)	(3.9)	(14.8)	(1.6)	(3.0)
			4	40	23	64	3	17
				(9.1)	(5.2)	(14.6)	(0.7)	(3.9)
			5	40	27	66	4	14
				(9.1)	(6.2)	(15.0)	(0.9)	(3.2)
		$\mathcal{M}_t$	3	0	20	0	61	318
				(0.0)	(1.7)	(0.0)	(5.1)	(26.5)
			4	0	0	0	59	341
				(0.0)	(0.0)	(0.0)	(4.9)	(28.4)
			5	0	0	0	60	340
				(0.0)	(0.0)	(0.0)	(5.0)	(28.4)
	0.150	$\mathcal{M}_b$	3	281	39	0	31	49
				(23.4)	(3.2)	(0.0)	(2.6)	(4.1)
			4	306	67	0	8	19
				(25.5)	(5.6)	(0.0)	(0.7)	(1.6)
			5	320	72	0	3	5
				(26.7)	(6.0)	(0.0)	(0.2)	(0.4)
		$\mathcal{M}_{bh}$	3	93	14	0	281	12
				(7.8)	(1.2)	(0.0)	(23.4)	(1.0)
			4	15	5	0	380	0
				(1.2)	(0.4)	(0.0)	(31.7)	(0.0)

Continued on next page

Table S4 continued...

N	Expected Pr(Encounter)	Data-generating model	Lists	$\mathcal{M}_b$	$\mathcal{M}_{bh}$	$\mathcal{M}_h$	$\mathcal{M}_{ht}$	$\mathcal{M}_t$
			5	3	0	0	397	0
				(0.2)	(0.0)	(0.0)	(33.1)	(0.0)
		$\mathcal{M}_h$	3	0	0	354	46	0
				(0.0)	(0.0)	(29.5)	(3.8)	(0.0)
			4	0	0	373	27	0
				(0.0)	(0.0)	(31.1)	(2.2)	(0.0)
			5	0	0	381	19	0
				(0.0)	(0.0)	(31.8)	(1.6)	(0.0)
		$\mathcal{M}_{ht}$	3	38	21	54	9	16
				(8.4)	(4.7)	(12.0)	(2.0)	(3.5)
			4	53	28	49	3	28
				(11.8)	(6.2)	(10.9)	(0.7)	(6.2)
			5	46	22	57	5	22
				(10.2)	(4.9)	(12.6)	(1.1)	(4.9)
		$\mathcal{M}_t$	3	1	18	0	55	326
				(0.1)	(1.5)	(0.0)	(4.6)	(27.2)
			4	0	1	0	62	337
				(0.0)	(0.1)	(0.0)	(5.2)	(28.1)
			5	0	1	0	62	337
				(0.0)	(0.1)	(0.0)	(5.2)	(28.1)
	0.200	$\mathcal{M}_b$	3	295	55	0	14	36
				(24.6)	(4.6)	(0.0)	(1.2)	(3.0)
			4	328	65	0	0	7
				(27.3)	(5.4)	(0.0)	(0.0)	(0.6)
			5	334	66	0	0	0
				(27.8)	(5.5)	(0.0)	(0.0)	(0.0)
		$\mathcal{M}_{bh}$	3	23	10	0	367	0
				(1.9)	(0.8)	(0.0)	(30.6)	(0.0)
			4	0	0	0	400	0
				(0.0)	(0.0)	(0.0)	(33.3)	(0.0)
			5	0	0	0	400	0
				(0.0)	(0.0)	(0.0)	(33.3)	(0.0)
		$\mathcal{M}_h$	3	0	0	348	52	0
				(0.0)	(0.0)	(29.0)	(4.3)	(0.0)
			4	0	0	352	48	0
				(0.0)	(0.0)	(29.3)	(4.0)	(0.0)
			5	0	0	367	33	0
				(0.0)	(0.0)	(30.6)	(2.8)	(0.0)
		$\mathcal{M}_{ht}$	3	35	20	54	4	19
				(8.4)	(4.8)	(13.0)	(1.0)	(4.6)
			4	39	22	55	7	14
				(9.4)	(5.3)	(13.3)	(1.7)	(3.4)

Header note: columns $\mathcal{M}_b$ through $\mathcal{M}_t$ are under "AIC-best estimating model".

Continued on next page

Table S4 continued...

N	Expected Pr(Encounter)	Data-generating model	Lists	AIC-best estimating model				
				$\mathcal{M}_b$	$\mathcal{M}_{bh}$	$\mathcal{M}_h$	$\mathcal{M}_{ht}$	$\mathcal{M}_t$
			5	47	23	56	7	13
				(11.3)	(5.5)	(13.5)	(1.7)	(3.1)
		$\mathcal{M}_t$	3	2	5	0	63	329
				(0.2)	(0.4)	(0.0)	(5.3)	(27.4)
			4	0	0	0	69	331
				(0.0)	(0.0)	(0.0)	(5.8)	(27.6)
			5	0	0	0	68	332
				(0.0)	(0.0)	(0.0)	(5.7)	(27.7)
