# Supplementary material for: Comparative performance of multiple-list estimators of key population size
Source: PLOS Glob Public Health. Author manuscript; Available in PMC 2023 Mar 10. (PMC9345571; doi:10.1371/journal.pgph.0000155)
Supplement: Supplementary file 2 [file NIHMS1791057-supplement-S1_Text.pdf]

S1 Text. Supplemental Methods and Results

Expectation of probabilities of first encounters

Let $p_1(k)$ denote the probability of first encounters with unique population members in list/sampling encounter k, and let p denote the per-list encounter probability. Before any sampling there are N unencountered population members. By definition, Np members appear on the first list, leaving $N - Np = N(1-p)$ members who have not yet been encountered. Again by definition, $N(1-p)p$ newly encountered members will appear on the second list, leaving $N(1-p) - N(1-p)p = N(1-p)^2$ who have not yet been encountered after the second listing. Successive iteration yields $p_1(k) = p(1-p)^k$. The expectation of $p_1(k)$ with respect to the Beta distribution having parameters α and β is defined as

$$\mathrm{E}[p_1(k)] = \int_0^1 p(1-p)^k \frac{p^{\alpha-1}(1-p)^{\beta-1}}{\mathrm{B}(\alpha,\beta)} dp,$$

where

$$\mathrm{B}(\alpha,\beta) = \frac{\Gamma(\alpha)\Gamma(\beta)}{\Gamma(\alpha+\beta)}$$

and where $\Gamma(x)$ is the Gamma function of x. Algebraic simplification of the integrand yields

$$\mathrm{E}[p_1(k)] = \frac{1}{\mathrm{B}(\alpha,\beta)} \int_0^1 p^{(\alpha+1)-1}(1-p)^{(\beta+k-1)-1} dp$$

which is recognizable as

$$\mathrm{E}[p_1(k)] = \frac{\mathrm{B}((\alpha+1),(\beta+k-1))}{\mathrm{B}(\alpha,\beta)}$$

giving

$$\mathrm{E}[p_1(k)] = \frac{\Gamma(\alpha+\beta)\Gamma(\alpha+1)\Gamma(\beta+k-1)}{\Gamma(\alpha)\Gamma(\beta)\Gamma(\alpha+\beta+k)}.$$

Sensitivity of Bayesian model-averaging estimates to the prior specification of the maximum population size

The sensitivity of population size estimation (PSE) to the prior specification of the maximum population size N_{max} using Bayesian model-averaging of log-linear models (LLM-BMA), implemented by the dga R package, was evaluated by computing three replicate estimates from the $\mathcal{M}_{bht}$ data-generating process for three- and five-list sampling. For a population of 10,000 members, N_{max} was specified as 50,000, 100,000 and 500,000. The resulting estimates of population size were constant across all values of N_{max} (S1 Fig. 1). That is, LLM-BMA estimates are insensitive to N_{max} as long as N_{max} is at least 50 times larger than N.

Sensitivity of Bayesian nonparametric latent-class estimation to the specification of the initial number of latent classes

The sensitivity of PSE to the prior specification of the maximum number of latent classes using Bayesian nonparametric latent class modeling (LCM), as implemented by the LCMCR R package, was evaluated by computing estimates from the $\mathcal{M}_{bht}$ data-generating process for three- and five-list sampling from a population of 10,000 members. Theoretically, the number of latent classes could equal the number of population members. Estimates became observably stable with the default value of five latent classes (S1 Fig. 2). There is little computational penalty to prior specification of 10 latent classes, which might better assure stable estimation.

Sensitivity of Bayesian nonparametric latent-class estimation to the hyperprior parameters for the stick-breaking parameter

The stick-breaking parameter which enforces parsimony in the estimated number of latent classes follows a Gamma prior distribution having shape and rate parameters [1]. The sensitivity of PSE to those parameters was evaluated by computing three replicates LCM estimates from the $\mathcal{M}_{bht}$ data-generating process for three- and five-list sampling from a population of 10,000 members. The default value for both the shape and rate parameters in the LCMCR R package is 0.25. The shape and rate parameters were assigned values of 0.025, 0.25 and 1.00. LCM estimates varied little over that range of prior parameters (Fig. 3).

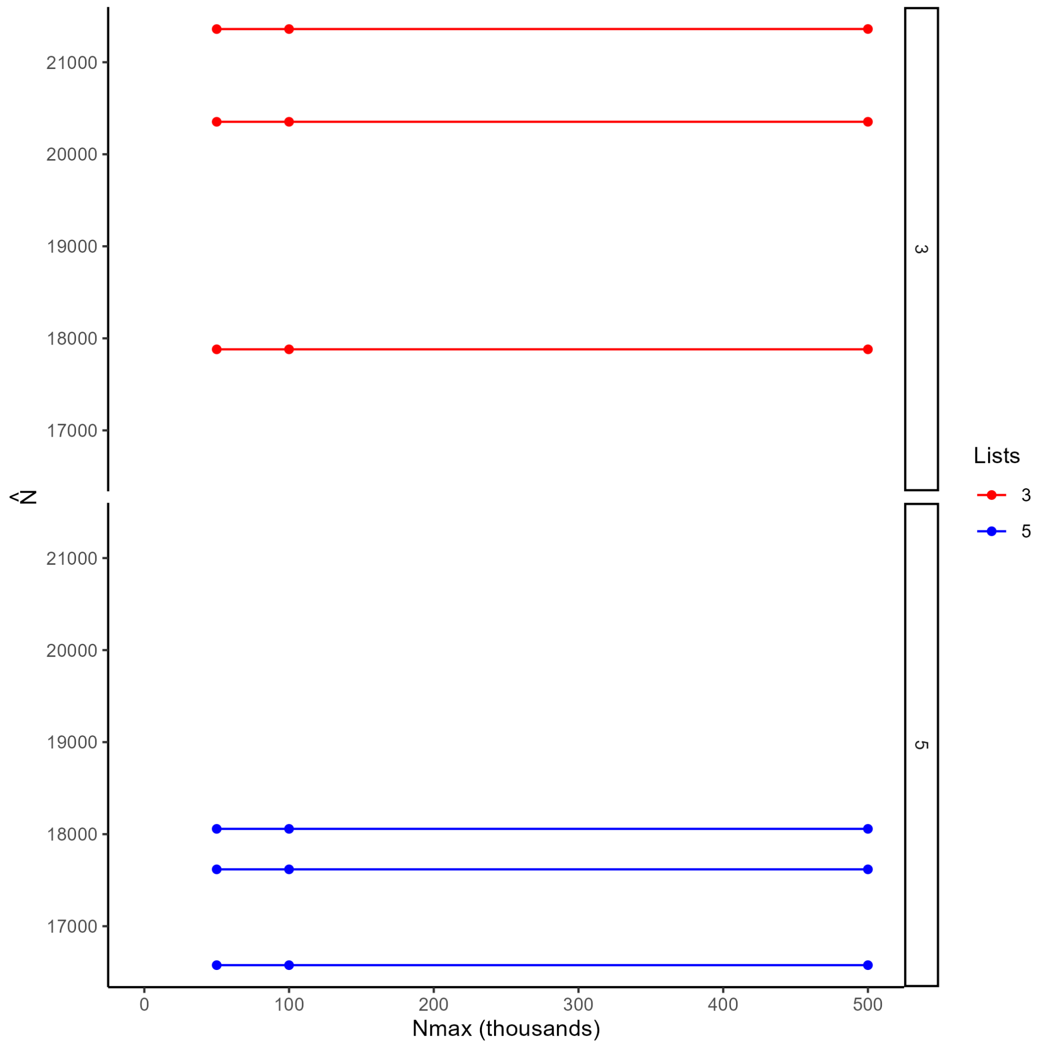

S1 Figure 1: Sensitivity of estimates of population size $\hat{N}$ from Bayesian model averaging of log-linear models to the prior specification of the maximum population size N_{max} from 3- and 5-list sampling from a population of 10,000 members.

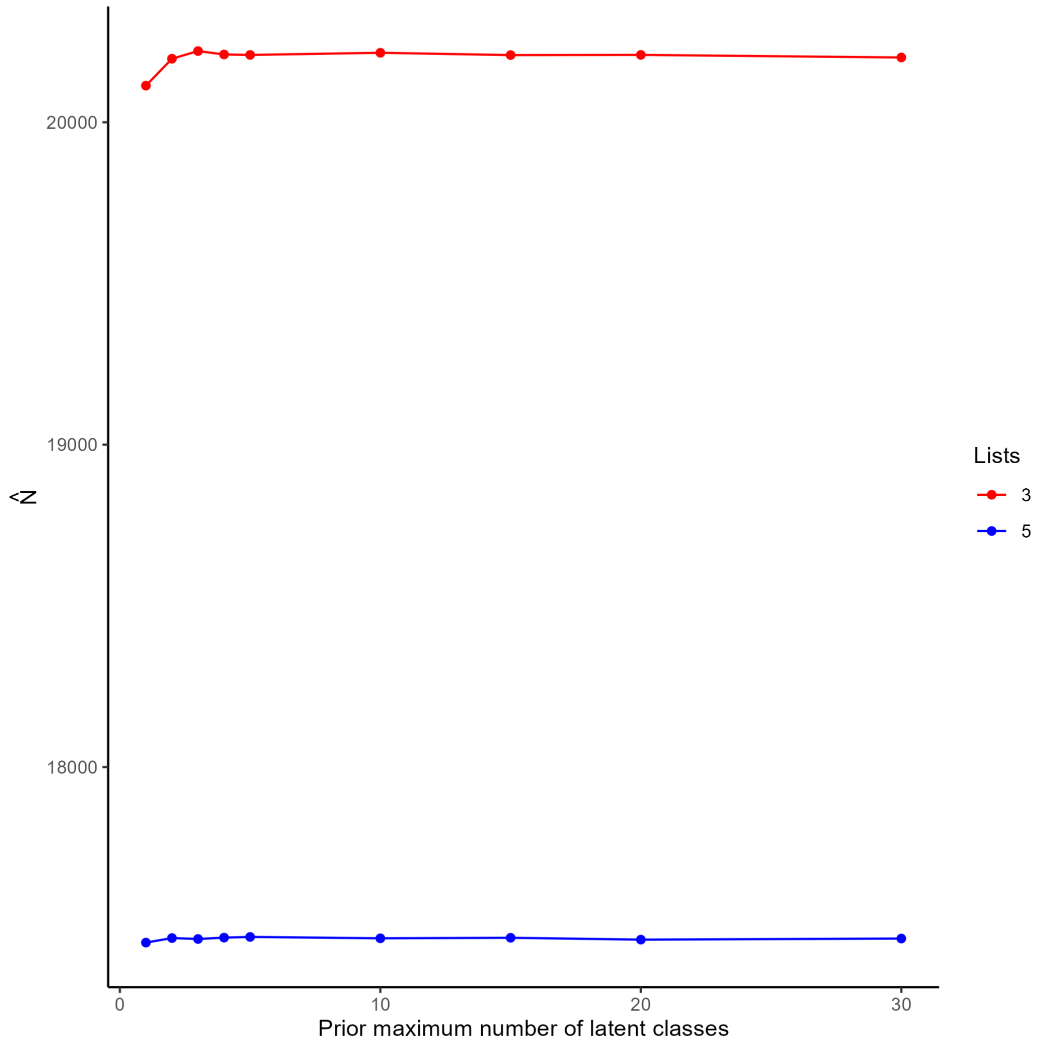

S1 Figure 2: Sensitivity of estimates of population size $\hat{N}$ from Bayesian nonparametric latent-class modeling to the prior specification of the maximum number of latent classes from 3- and 5-list sampling from a population of 10,000 members.

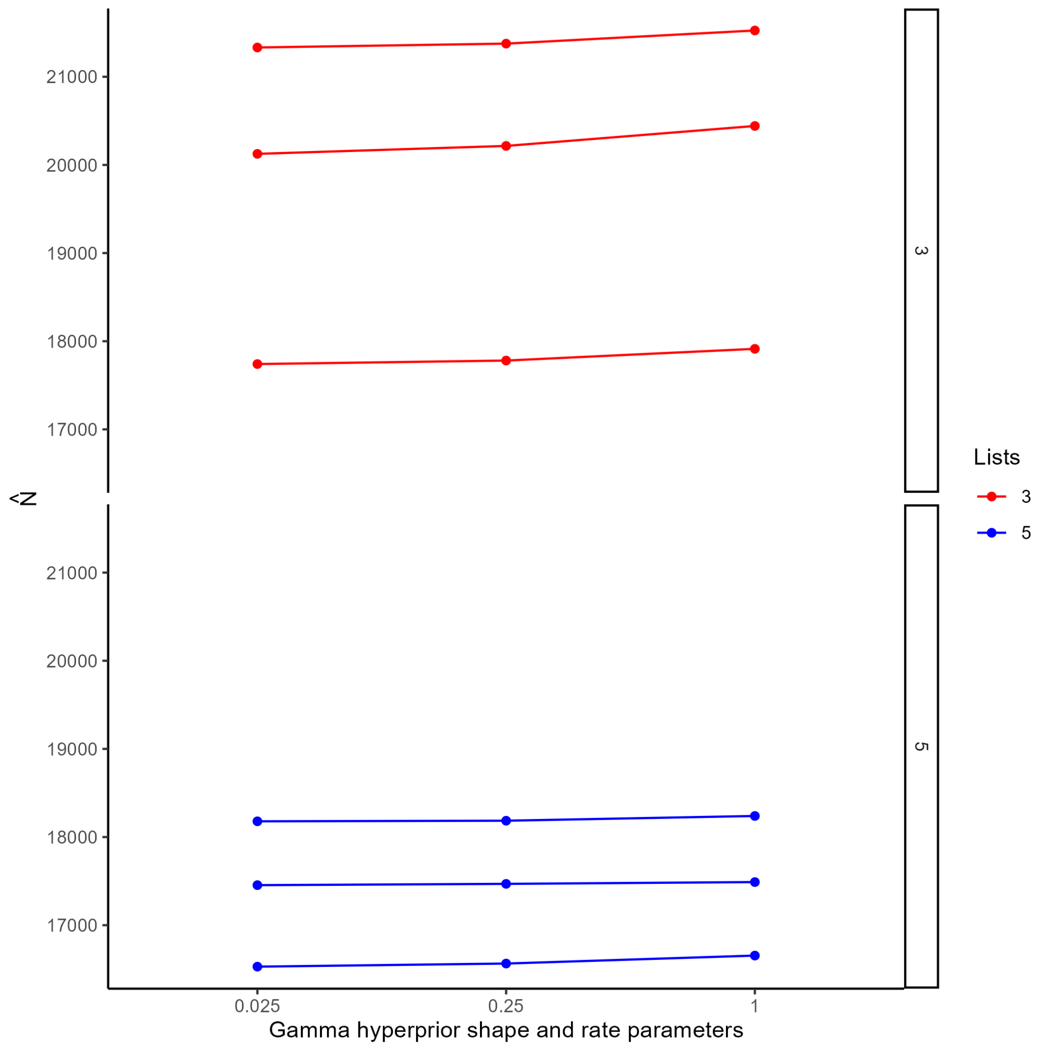

S1 Figure 3: Sensitivity of estimates of population size $\hat{N}$ from Bayesian nonparametric latent-class modeling to the Gamma-prior shape and rate parameters for the stick-breaking parameter from 3- and 5-list sampling from a population of 10,000 members.

Bibliography

[1] Manrique-Vallier D. Bayesian population size estimation using Dirichlet process mixtures. Biometrics. 2016;72(4):1246–1254. doi:10.1111/biom.12502.
